# A Systematic Review of Translation and Cross-Cultural Adaptation of Instruments for the Selection of Assistive Technologies

**DOI:** 10.1155/2018/4984170

**Published:** 2018-11-01

**Authors:** Bárbara Iansã de Lima Barroso, Cláudia Regina Cabral Galvão, Luiz Bueno da Silva, Selma Lancman

**Affiliations:** ^1^Graduate Student of the Graduate Program in Rehabilitation Sciences, Faculty of Medicine FMUSP, University of São Paulo, São Paulo, SP, Brazil; ^2^Professor of the Postgraduate Program in Production Engineering, Federal University of Paraíba UFPB, João Pessoa, PB, Brazil; ^3^Professor of the Postgraduate Program in Rehabilitation Sciences. Faculty of Medicine FMUSP, University of São Paulo, São Paulo, SP, Brazil

## Abstract

This article presents a systematic review, for which research was carried out in the following electronic databases: Web of Science, Scopus, PubMed, and ERIC, in which several articles were found (*n* = 560). The results were analyzed based on the information obtained in the titles and abstracts, and the remaining studies were read in full for the analysis. The aim of this article was to identify the different questionnaires used for selecting items of assistive technology, describe and evaluate the translation and transcultural adaptation procedures, characterize the thematic domains of each resource, evaluate the cultural adaptation process adopted, and describe the psychometric properties. Data extraction and evaluation of the methodological quality of the eligible studies were performed in accordance with the COSMIN verification list with a checklist of 4 points. The publication of systematic review studies and others that synthesize research results is fundamental to provide support for change in the behavior of professionals in the field of health, and not only to access the available literature but also to incorporate this information into daily clinical practice. The results of this review could provide subsidies that would enable the planning, execution, identification, and choice of specific instruments for a determined study within the context of assistive technology, with a view to helping researchers and health professionals in clinical and investigatory practices.

## 1. Introduction

According to the World Health Organization (WHO, 2008) [1], approximately 10% of the world population has some type of deficiency, and, of this total, only 3% of them require rehabilitation and receive some aid in underdeveloped countries. In Brazil, disability affects 23.9% of the population (IBGE, 2010) [2].

In 2001, the WHO approved the classification system—International Classification of Functioning, Disability and Health (ICF)—which is a tool for understanding human functionality and incapacity, recommended for identifying structural and environmental conditions and personal characteristics that interfere with functionality [3]. This has changed the approach based on diseases and prioritizes focus on functionality and autonomy [4].

Considering the functions of the human body, autonomy mainly involves each individual's capacity to care for themselves: performing tasks enabling them to adapt to situations and take responsibility for their own acts, thereby improving their mobility, communication, and autonomy; reinforcing their social participation, professional productivity, and sense of control of their own lives [3].

Measurement instruments were used to obtain standardized data that enabled comparison of results between different populations [5], and they are an economic and effective way to acquire reliable and valid measurement results [6]. These instruments transform subjective measurements into quantifiable objective data that can be analyzed, enabling verification of the impacts on health intervention processes, and comparisons would therefore require fewer financial resources.

Researchers conducting exploratory researches have to decide whether to use previously developed instruments or to construct one specifically suited to their studies. As the process of creating a new instrument is complex, and provided that there are validated instruments for assessing the same phenomenon, the process of translation and adaptation to the desired culture is recommended [7].

Cook and Polgar [8] defined assistive technology as the science that deals with the application of any item, part, equipment, service, or product system of high or low technology that is commercially acquired, modified, or custom-made, and which is used for increasing, maintaining, promoting, or developing the functional capacity of disabled people.

Compared with other North American and European countries, Brazil has few instruments to help researchers and professionals in the field of rehabilitation with the process of prescribing, indicating, and selecting assistive technology items. Evaluation and rehabilitation processes have limitations, since achieving their real objectives is known to depend on the adequate selection and use of suitable instruments. To use the evaluation instruments available in the literature in assistive technology and in other areas of health, they must be suited to the purpose by following the guidelines for cultural adaptation, translation, and test measurement properties [4, 9–11]. However, to use in a new different country, culture, and/or language, cultural adaptation requires a methodology making it possible to achieve: equivalence [10], solving cultural and idiomatic differences, and a process for standardization and validation of the measurement properties [4, 12, 13].

Cultural adaptation must result in a reliable valid instrument, similar to the original, capable of being used as a reference in clinical research and replicated in different cultures; i.e., a tool that can be used to compare results obtained in different countries [5]. The influential cultural, psychosocial, financial, physiological, and biomechanical factors of distinct cultures must be sufficiently understood to help rehabilitation professionals make decisions during therapeutic processes [9, 11].

In this article, we proposed to conduct a systematic review to identify different questionnaires used for selecting assistive technology items, describe and evaluate translation and transcultural adaptation procedures and evaluate the process adopted, characterize the thematic domains of each instrument and describe the psychometric properties found in the studies [11, 12]. This review may result in subsidies for planning, identifying, and choosing specific instruments for a determined study, within the context of assistive technology, and be helpful to researchers and health professionals in their clinical and investigatory practices.

## 2. Materials and Methods

This bibliographic research was defined as a systematic review. To conduct this research, the authors adopted the Preferred Reporting Items for Systematic Reviews and Meta-Analyses (PRISMA) criteria. According to Liberati et al. [14], PRISMA assists systematic review writing and is essential for precisely and reliably summarizing proofs of the efficacy and safety of health interventions.

The literature search was performed during two periods between 19th July, 2016 and 31st August, 2017 (the date it was updated), and there was a manual addition of them between 6th and 12th of July, 2018, updating the files of this review.

According to the PRISMA-P committee guidelines [15], this research was registered with the International Prospective Register of Systematic Reviews (PROSPERO) on 19th July, 2016 and updated on 31st August, 2017. Registration number CRD42016043065.

Although the PRISMA recommendations were primarily developed for textual presentation of systematic reviews of interventions, studies of prognosis [16] and clinical diagnosis [17], transcultural adaptations [18], measurement properties [19], and others [11, 20] have used them extensively.

### 2.1. Search Strategy

The following electronic databases were sequentially searched for articles, without any language restriction: Web of Science, Scopus, PubMed/MEDLINE, and the Educational Resources Information Center (ERIC), with terms used based on Medical Subject Headings (MeSH) descriptors. The literary bibliography was searched using the following descriptors: “Assistive technology” AND “Cross-cultural adaptation” OR “Assistive technology device” AND “Translation” OR “Validation” AND “Successful Rehabilitation” OR “Assistive technology selection”. Variations in the descriptors served to find a broader range of significant results for this research.

### 2.2. Inclusion and Exclusion Criteria

The inclusion criteria adopted were as follows: articles must be indexed in the selected data bases, be available in the free form of the text, written in any language, they must not have data filters, be without restrictions relative to the manuscript's place of origin, and without exclusion criteria due to language in the translation and cultural adaptation process.

Due to the inclusion and exclusion criteria employed in our systematic review, a small number of articles [21–25] were not included in our overall sample (*n* = 560). Therefore, the findings of these articles were not included in our final analysis. Given our large article sample, we do not anticipate that this omission caused any substantial change in our results. Yet, to mitigate this issue, we intend to include these articles in a future review to measure their specific impact.

### 2.3. Eligibility Criteria—Selection of Studies

The studies considered and fulfilled the following criteria: (1) quantitative cross-sectional researches and (2) studies investigating the translation and cross-cultural adaptation process of instruments for selecting assistive technology for disabled people.

### 2.4. Data Extraction and Quality Evaluation

**Figure 1:**
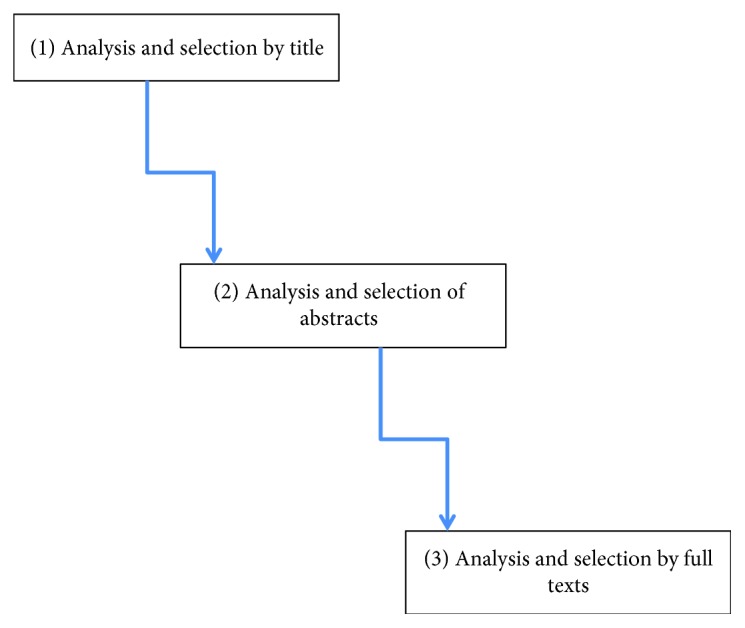
The selection process hierarchically included the following strategies.

Two researchers screened paper titles and abstracts and applied the eligibility criteria. The papers that did not meet the eligibility criteria were excluded. The selection process hierarchically included the following strategies: (1) analysis and selection by title, (2) analysis and selection of abstracts, and (3) analysis and selection by full texts (Figure 1).

One reviewer extracted the data independently at the time of the search and again after the data analysis. Subsequently, a second researcher independently analyzed and reviewed the data. Consequently, no third reviewer or final arbitration was required. One researcher conducted data extraction of all eligible studies, which were summarized in table depicting their descriptive characteristics.

Data was extracted with the purpose of finding out how the translation, cross-cultural adaptation procedures, and all measurement properties of each study included were performed. Moreover, Consensus-based Standards for selecting health Measurement Instruments (COSMIN) [4] were used to classify the translation, cross-cultural adaptation procedures, and psychometric measurement properties, respectively. The evidence of validity was analyzed according to the following criteria [4, 5, 10, 13, 14, 17, 20]: for internal consistency, Cronbach's alpha >0.70 (ideal) [4, 5, 20, 26], and also in accordance with the COSMIN guidelines [4, 12].

### 2.5. Consensus-Based Standards for Selecting Health Measurement Instruments (COSMIN)

In the literature, the taxonomy of measurement properties presented a great variety; however, in this study, the authors used the definitions proposed by COSMIN to evaluate the psychometric properties of instruments. The authors chose to describe the test performed, results and criteria for evaluating the psychometric properties of instruments developed, adapted, and validated [4, 5, 13, 27].

A 4-point classification scale was used to mark and classify the methodological quality of each measurement property: (A) internal consistency, (B) reliability, (C) measurement error, (D) content validity, (E) structural validity, (F) hypothesis tests, (G) cross-cultural validity, (H) criterion validity, and (I) responsiveness. Each box was finally classified by the lowest score attributed to any of the items [4, 13, 28]. Apart from the above-mentioned boxes, another field had to be completed for each measurement property, with the aim of identifying the population's clinical-epidemiological profile, analyzing mean age, distribution by gender, disease characteristics, country of origin, and language [4, 28–30].

## 3. Results

**Figure 2:**
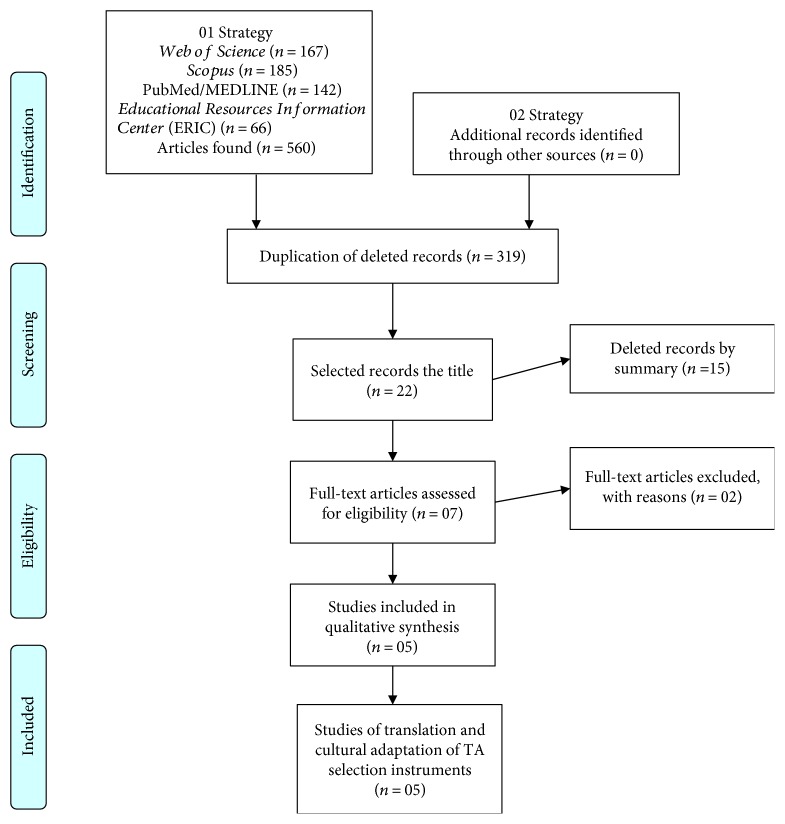
PRISMA 2009 Flow Diagram—selection process for the studies included in the analysis.

In the initial research, 560 potentially eligible studies were identified in the systematic review (Figure 2). Of these, 319 were excluded because they were duplicates; 241 remained, of which 210 were discarded after reading the title and abstract, leaving 22 studies. Of these, two were excluded because they were not available in full. Thus, the 20 remaining studies were read in full, after which 15 were discarded because they did not fit in with the eligibility criteria. Finally, only five researches were included in the qualitative synthesis because they fulfilled the inclusion criteria.

The five studies of translation and cross-cultural adaptation evaluated after reading the full texts were the following: Quebec User Evaluation of Satisfaction with Assistive Technology (QUEST 2.0), translated and adapted from English into Brazilian Portuguese [31]; Psychosocial Impact of Assistive Devices Scale (PIADS), from English into Canadian French [32]; Quebec User Evaluation of Satisfaction with Assistive Technology 1.0 (QUEST1.0), from English into Danish [33]; Family Impact of Assistive Technology Scale, from English into Turkish [34]; and Quebec User Evaluation of Satisfaction with Assistive Technology (QUEST 2.0), from English into Chinese (Mandarin) and adapted to Taiwanese [35], as demonstrated in Table 1.

In Table 2, the translations and transcultural adaptations evaluated were presented according to the guidelines proposed by Mokkink et al.—COSMIN committee [4, 12].

### 3.1. Internal Consistency

The five instruments [31–35] were tested for internal consistency; however, only three [32, 34, 35] were classified as “excellent”, because they fulfilled the COSMIN requisites. The translation and validation of the Quebec User Evaluation of Satisfaction with Assistive Technology (QUEST 2.0) [31], translated from English into Portuguese, was classified as “good”, because it had partially completed the checklist of the guidelines. The methodological quality of the Quebec User Evaluation of Satisfaction with Assistive Technology (QUEST 1.0) [33] was “fair” in all the subclassification of (Box A).

The intraclass correlation coefficient (ICC) and Cronbach's alpha (*α*) were used to evaluate the reliability and internal consistency in the majority of the studies selected in this review [31, 32, 34, 35], and *ρ* for Cronbach's alpha (*α* = 0.70) was adopted as the minimum acceptable value [32].

One research [35] also used Cronbach's alpha relative to the ICC values; four studies performed the test [31, 32, 34, 35], and the value ≥0.70 was appropriately obtained [35]. The ICC demonstrated an error of measurement and agreement, as the relationship between the true and the observed variance. There was no consensus regarding the value the ICC indicated as an acceptable degree of reliability; however, the majority of the authors agreed that to appropriate values, at least (≥0.70) was required [11, 26, 30].

The study “Translation and validation of the Quebec User Evaluation of Satisfaction with Assistive Technology” (QUEST 2.0) [31], translated from English into Brazilian Portuguese, and the “Translation, cross-cultural adaptation, and content validation of the QUEST 1.0” [33] did not present the reproducibility/reliability tests of the instruments.

In order to know more about the ICC of QUEST 2.0 [31], the authors of this review sent e-mails to the main authors, asking about the application of the ICC and/or Kappa to measure the internal consistency and/or other forms for evaluating the point at which individuals could be distinguished from one another in spite of measurement errors. The author sent her dissertation data, informing that she used test (a) to evaluate the internal consistency of the instrument, which indicated the level of the instrument, or of the questions of which it was composed, and that it was sensitive in capturing the change in values after removing each item [36]. For this systematic review, this data was not tabulated because, according to [4], it was not available in the article analyzed [31].

### 3.2. Reliability

The reliability of all the instruments was tested. The Quebec User Evaluation of Satisfaction with Assistive Technology (QUEST 1.0) [33] was classified as “poor” because it used a sample below the number (*n* = 10) indicated and did not apply the statistical methods indicated.

The test-retest ICC reliability of the Turkish version of the Family Impact of Assistive Technology Scale (FIATS) [34], translated and validated from North American English into Turkish (*n* = 55), obtained the validity and reliability score of 0.931 (95% 0.881–0.960), with an excellent estimate. The Psychosocial Impact of Assistive Devices Scale (PIADS) [32] instrument, translated and validated from North American English into Canadian French (*n* = 304), achieved good stability in the ICC (0.77–0.90) and internal consistency (0.75–0.94) tests. For the Quebec User Evaluation of Satisfaction with Assistive Technology (QUEST 2.0) [31] instrument, translated and validated from North American English into Brazilian Portuguese (*n* = 121), the test-retest stability, which was performed two months later, was analyzed by the Spearman correlation test, demonstrating a high correlation (*ρ* > 0.6) for the majority of the items. These three instruments [31, 32, 34] were classified as “good”.

The Quebec User Evaluation of Satisfaction with Assistive Technology (QUEST 2.0) [35], translated from North American English into Chinese (mandarin) (*n* = 105), although it specifically achieved an ICC of (0.90, 0.97, 0.95), positive total scores of the device, services and T-QUEST, and good test-retest stability were classified as “good”.

Further, to the Taiwanese QUEST 2.0 [35], the exploratory factorial analysis revealed that the T-QUEST had a two-factor structure for the service device and construction of user satisfaction, which constituted 53.42% of the variance explained.

### 3.3. Measurement Error

Measurement error was verified in all the instruments, and only three were considered “excellent”—PIADS [32], FIATS [34], and QUEST 2.0—translated from North American English into Chinese (mandarin) [35].

Further, to the Quebec User Evaluation of Satisfaction with Assistive Technology (QUEST 2.0) [31], despite the appropriate sample number (*n* = 121) indicated by the guidelines (≥100) [4, 12], the statistical quality was classified as “fair”. Whereas, the Quebec User Evaluation of Satisfaction with Assistive Technology (QUEST 1.0) [33] was classified as “poor”, due to the sample (*n* = 10) and lack of statistical tests.

### 3.4. Content Validity

The content validity was analyzed in the five articles; however, there was a difference related to the COSMIN checklist score. The manuscript [33] was classified as “fair”, due to small methodological failures in the study presentation. The researches [31, 32] were classified as “good”, and the studies [34, 35] as “excellent”; therefore, they achieved positive points in the entire checklist.

### 3.5. Structural Validity

After verification, the article [33] was classified as “poor”, because it presented no statistical resources. The research [31] was classified as “good” and the studies [32, 34, 35] as “excellent”. These results represented excellent structural validity for all the questions tested in these instruments.

### 3.6. Cross-Cultural Validity

The field for transcultural validity received the highest positive value among the items analyzed. Four instruments were adapted to their respective cultures: Brazilian [31], Canadian [32], Turkish [34], and Chinese [35], according to the method recommended [4, 12] and were classified as “excellent”.

The Quebec User Evaluation of Satisfaction with Assistive Technology (QUEST 1.0) [33] transcultural adaptation was classified as “good”, although its sample size was inadequate (<50).

### 3.7. Criterion Validity

The Quebec User Evaluation of Satisfaction with Assistive Technology (QUEST 1.0) [33] was classified as “poor”, due to the sample's size (<50), lack of statistical analyses, and other measures of interpretability, with the clinically important minimum difference.

The ceiling and floor effects, reflecting interpretability of the questions, were verified in the Quebec User Evaluation of Satisfaction with Assistive Technology (QUEST 2.0) instrument [31], which was classified as “good”. The reliability or internal consistency of the instrument items was tested by Cronbach's alpha coefficient for each factor, for each item removed, and for total score. The Spearman correlation was used to analyze precision during the test-retest procedure, showing a relationship between the first and second applications of the instrument, thus corroborating Nunnally's psychometric theory [31, 37], which recommends a minimum of ten individuals for each existent item in a scale. In this research, the sample consisted of (*n* = 121).

The Psychosocial Impact of Assistive Devices Scale (PIADS) [32], Family Impact of Assistive Technology Scale (FIATS) [34], and Chinese Quebec User Evaluation of Satisfaction with Assistive Technology (QUEST 2.0) [35] instruments were classified as “excellent”. Their hypotheses and statistical methods were formulated and tested.

### 3.8. Responsiveness

In this item, the article [33] was classified as “poor”, the research [31] as “good”, and the studies [32, 34, 35] as “excellent”.

## 4. Discussion

The World Health Organization (WHO) recommends the translation and cross-cultural adaptation of the existent instruments available for selection, into different languages and cultures, thereby favoring communication between different researchers and the comparison of data obtained at an international level [38].

Standardized assessments are the tools that facilitate the process of prescribing and monitoring the use of assistive technology resources in occupational therapy and other professions. They favor the understanding of the demands of the subjects and allow professionals to identify which important areas will be considered in an evaluation process, and the degree of satisfaction and functional improvement after a certain period of use of the AT device [39].

The use of a standardized evaluation allows the foundation of new research and the development of products, as well as the verification of its impact in the formulation of public policies and the identification of the sustainability of rehabilitation programs in assistive technology. The results produced, when valid and reliable, favor the credibility of the professional area for the generation of the measured results and their influences [39–42].

The systematic review of the properties of measurement is a process in which the content and properties of measurements by the measurement instruments are critically and comparatively evaluated in detail. The systematic reviews of these properties are useful tools for selecting a measurement instrument for a certain purpose [26].

Five different studies [31–35] were found, of which three concerned the same instrument of assistive technology selection: the Quebec User Evaluation of Satisfaction with Assistive Technology (QUEST)—one, in version 1.0, and two, in version 2.0. In all of the five articles, some of their measurement properties were tested. However, the use of an instrument in a new country or a new culture—albeit within the same country—requires a method that guarantees equivalence between the original versions. Therefore, the instrument content must undergo cross-cultural adaptation and validation to guarantee maximum faithfulness [4, 6, 10, 12, 13, 33].

The COSMIN initiative was created in 2005, which was inspired by the lack of clarity in the literature about terminologies and definitions of measurement properties. A large number of instruments for the measurement of results—many of them similar—are used for measuring the same mechanisms developed with focus on the same population of patients, with criteria that are not always coherent with the culture and the need for the local population to be researched and/or attended [4, 12].

The COSMIN checklist may be used to evaluate the methodological quality of studies regarding the properties of measurement of health status or to compare their properties in various measurement instruments during a systematic review. The methodological quality of the high- and low-quality studies selected must be analyzed and possible bias indicated [4, 43].

As far as the stages of translation and cross-cultural adaptation proposed by [4, 12] are concerned (these are presented in Table 2), all the measurement properties were checked. For Coster and Mancini [7]and Guillemin et al. [10], the maximum equivalence between the original instrument and its translated and adapted version must guide the entire process to prevent (frequently subtle) forms of interpretation, causing distortion in both the process of using the instrument and in the values obtained [37].

Generally speaking, the most used test was the intraclass correlation coefficient (ICC), which is calculated based on a ratio of variance. Four studies used the test [31, 32, 34, 35], and the value ≥0.70 was obtained in an appropriate manner. They were classified as “good” [4, 12, 15]. No instrument completely tested all the properties of the measure of reliability, and none were able to achieve the classification “excellent” (Table 2).

The criteria most tested, based on the COSMIN classification, were those of internal consistency, content validity, and cross-cultural validity. Internal consistency is the interrelationship between the components of the questionnaires, content validity includes face validity, and cross-cultural validity is the capacity of an instrument to obtain similar results with different individuals and evaluators. This may be evaluated by the reliability and agreement of the construct [4, 12].

All the instruments performed the pretest [31–33, 35] or pilot-test (as it was denominated in the study [34]). The majority of the studies adequately described the sample [31–33, 35], and only one [34] was not in agreement with COSMIN [4, 12, 13].

To generalize the results of the transcultural adaptation process, the COSMIN checklist recommends that the participants involved in the pretest should be clinically and epidemiologically described in terms of age, sex, characteristics of the disease/deficiency, and source of participant recruitment (hospital, clinic, rehabilitation center, university, therapeutic community, etc.).

The authors also observed that in some instruments [31–34] the retro-translation was done by two different person, who were natives of the original language of the instrument to be translated. The instrument [35] did not mention the quantity of translators and related that the process was performed by a committee [4, 12, 43, 44].

It is recommended that the translation procedure should be done by two different person in an independent manner. Comparison between two different translations assures the precision of the original version, with a more appropriate process, and maintenance of the semantic equivalence. It is important for the translators or the team not to communicate with one another about the work during the translations that will be compared afterwards [12, 13, 44].

Although the searches were conducted in the most used databases, using all the 27 items of the checklist included in the report of the systematic review and meta-analysis [12, 13, 18], some studies may not have been included in this systematic review.

## 5. Conclusion

This systematic review presented assistive technology instruments that have undergone the process of translation and cross-cultural adaptation. The publication of systematic reviews that synthesize the results of research is fundamental for bringing about a change in practices and behaviors among health care professionals. As such, the present review may provide subsidies for planning, identifying, and choosing specific instruments for a determined objective, within the context of the assistive technology field, while also being helpful for researchers and health professionals in their clinical and investigatory practices. This transformation requires that these professionals not only consult the available literature but also incorporate the information and knowledge provided by it into their daily clinical practice.

QUEST may be considered as the instrument that is most translated and adapted to other cultures and appropriate for use, when compared with other questionnaires tested for the same purpose. This happens because QUEST's properties of measurement were properly tested and most stages of cultural adaptation were performed.

It is important to emphasize, though, that due to the inclusion and exclusion criteria employed in our systematic review, a small number of articles (five) about PIADS were not included in our overall sample (*n* = 560). As such, it is possible that, because of this involuntary omission, some relevant findings may have not been included in the analysis performed in our review. Consequently, a future study will have to measure the impact of these missing papers. Yet, given our large article sample and our adopted strategy for decreasing the number of the studies in favor of assuring the quality of the results, we do not anticipate a priori that this omission may have caused any substantial change in our results and conclusions.

Finally, we want to mention that our study may have limitations related to a few types of assistive technology instruments, because our research was limited to questionnaires involving a limited set. Therefore, we suggest that further reviews should be conducted and that a meta-analysis should be included, which will present evidence and characteristics of different assistive technology instruments.

## Figures and Tables

**Table 1:** Translation and cross-cultural adaptations [4, 6, 10, 12].

Authors/year	Assistive technology tools	Language	Sample	Translation	Synthesis	Back translation	Expert committee review	Pretesting
de Carvalho et al. [31]/2014.	Quebec User Evaluation of Satisfaction with Assistive Technology (QUEST 2.0)	From English to Brazilian Portuguese	121	+	+	+	+	+

Demers et al. [32]/2002.	Psychosocial Impact of Assistive Devices Scale (PIADS)	From English to French Canadian	304	+	+	+	+	+

Brandt [33]/2005.	Quebec User Evaluation of Satisfaction with Assistive Technology (QUEST 1.0)	North American English to Danish	10	+	+	+	+	+

Bek et al. [34]/2012.	Family Impact of Assistive Technology Scale (FIATS)	North American English into Turkish	55	+	+	+	+	+

Mao et al. [35]/2010.	Quebec User Evaluation of Satisfaction with Assistive Technology (QUEST 2.0)	From English to Chinese (Mandarin)	105	+	+	+	+	+

+ = positive rating; − = negative rating; 0 = no information available; ? = unclear.

**Table 2:** Evaluation of the methodological quality of the included studies through the COSMIN checklist with 4-point rating scale: consensus-based standard for the selection of health measurement instruments [4, 12].

Instruments	Authors/year/language	Internal consistency (A)	Reliability (B)	Measurement error (C)	Content validity (D)	Structural validity (E)	Cross-cultural validity (G)	Criterion validity (H)	Responsiveness (I)
Quebec User Evaluation of Satisfaction with Assistive Technology (QUEST 2.0)	de Carvalho et al. [31]/2014. From English to Brazilian Portuguese	Good	Good	Fair	Good	Good	Excellent	Good	Good

Psychosocial Impact of Assistive Devices Scale (PIADS)	Demers et al. [32]/2002. From English to French Canadian	Excellent	Good	Excellent	Good	Excellent	Excellent	Excellent	Excellent

Quebec User Evaluation of Satisfaction with Assistive Technology (QUEST 1.0)	Brandt [33]/2005. North American English to Danish	Fair	Poor	Poor	Fair	Poor	Good	Poor	Poor

Family Impact of Assistive Technology Scale (FIATS)	Bek et al. [34]/2012. North American English into Turkish	Excellent	Good	Excellent	Excellent	Excellent	Excellent	Excellent	Excellent

Quebec User Evaluation of Satisfaction with Assistive Technology (QUEST 2.0)	Mao et al. [35]/2010. From English to Chinese (Mandarin)	Excellent	Good	Excellent	Excellent	Excellent	Excellent	Excellent	Excellent

The following property was not used in the study: box F. Hypotheses testing.
